# Culm Age and Rhizome Affects Night-Time Water Recharge in the Bamboo *Phyllostachys pubescens*

**DOI:** 10.3389/fpls.2017.01928

**Published:** 2017-11-10

**Authors:** Xiuhua Zhao, Ping Zhao, Zhenzhen Zhang, Liwei Zhu, Yanting Hu, Lei Ouyang, Guangyan Ni, Qing Ye

**Affiliations:** ^1^Key Laboratory of Vegetation Restoration and Management of Degraded Ecosystems, South China Botanical Garden, Chinese Academy of Sciences, Guangzhou, China; ^2^Guangdong Provincial Key Laboratory of Applied Botany, South China Botanical Garden, Chinese Academy of Sciences, Guangzhou, China; ^3^College of Geography and Environmental Sciences, Zhejiang Normal University, Jinhua, China

**Keywords:** *Phyllostachys pubescens*, night-time sap flow, age effect, water compensation, morphological features

## Abstract

Bamboo species—the only herbaceous trees—have unique structural and physiological characteristics that differ from those of other tree taxa. However, the role of night-time water use in bamboo is poorly understood and has rarely been investigated. We studied the day- and night-time sap flow response to culm age and rhizome structure in three age levels (juvenile, mature, and senescent) of *Phyllostachys pubescens* growing in the Nankun Mountain Natural Reserve, South China. We found that sap flow density and whole-tree hydraulic conductance decreased with culm age. After cutting of rhizome, the day-time sap flow and night-time water recharge decreased obviously. In addition, night-time water recharge accounted for the largest proportion (up to 30%) of total daily transpiration in normal senescent bamboos. Therefore, our study indicates that the connected rhizome system and night-time water recharge played a significant role in water compensation during the day and at night in bamboos. Night-time water recharge is especially critical to senescent bamboos, given their weaker transpiration due to the lower whole-tree hydraulic conductance, and consequently, they are more dependent on night-time water recharge for fulfilling their whole-day water consumption needs.

## Introduction

Night-time water recharge in tree trunks accounts for ∼10–50% of the total daily transpiration of a plant (Loustau et al., 1996; Goldstein et al., 1998; Steppe and Lemeur, 2004; Scholz et al., 2008; Carrasco et al., 2014). Thus, it plays an important role in relieving xylem hydraulic stress and controlling fluctuations in leaf water potential, in addition to regulating the stomatal openings and water status (Čermák et al., 2007; Meinzer et al., 2008). While some of this stored water is used in the development of plant structures and the maintenance of basic organizational functions, the remaining proportion is used to compensate for the water loss during daytime transpiration. This water store is recharged primarily via sap flow at night when no transpiration occurs (Zhao, 2010). According to previous studies, night-time sap flow not only ameliorates the water lost from water-storage tissues due to daily transpiration, but also enhances nitrogen uptake (Kupper et al., 2012; Rohula et al., 2014). It can keep the stomatal aperture open to increase CO_2_ diffusion capacity, optimize photosynthetic demand for the following morning (Barbeta et al., 2012), and transport O_2_ to anoxic xylem tissues (Sorz and Hietz, 2008; Gao et al., 2016). Night-time sap flow is a common hydro-physiological phenomenon observed in many dicot species and herbaceous plants (Goldstein et al., 1998; Gao et al., 2016), including bamboos (Zachary, 2009; Cao et al., 2012; Yang et al., 2015).

Owing to the absence of secondary growth, bamboo trees are unable to renew xylem to improve and enhance anti-cavitation mechanism. Tyloses and depositions in the transport system tubes gradually develop with age, which could eventually lead to the collapse of the water conduit system and even the death of the plant (Liese and Weiner, 1996). Therefore, they are forced to trade-off between satisfying their water needs and maintaining the long-term effectiveness of their transportation systems. Thus, it is likely that bamboo possesses adaptive mechanisms and strategies that differ from those of dicots. Studies have shown that bamboo species not only acquire and store water for the following day, but can also repair the vessel cavitation induced by the excessive tension caused due to strong daily transpiration using night-time sap flow. This ensures high water-delivery performance and long-term effectiveness of water- transport tissues (Cao et al., 2012). Tree form features are the most important factors influencing tree water use (Meinzer et al., 2005; McJannet et al., 2007). A study at Nankun Mountain in south China revealed that, in addition to morphological features, transport within culms of the bamboo *Phyllostachys pubescens* is greatly dependent on culm age, and that transpiration decreases significantly with increasing culm age. Bamboo has unique water-compensation properties due to the connectivity of different culms through the rhizome, which could relieve the high water demands of individual stems (Zhao et al., 2016). Thus, it remains unresolved whether bamboo species have an auxiliary water-transport mechanism that is not found in dicot tree species, and whether night-time sap flow is affected by culm age and rhizome.

To characterize and test the night-time sap flow and rhizome compensation function in bamboo, we chose *P. pubescens* as the model species, as it features a scattered-type rhizome that is convenient for the study of underground connections among culms and the rhizome water-compensation function. Moreover, *P. pubescens* forests are becoming increasingly important in the provision of ecosystem services, especially in terms of commercial applications, and are the most important source of non-wood forest products not only in China, but also possibly in the world (Song et al., 2016; Zhao et al., 2016). In China, stands of *P. pubescens* account for 70% of the country’s total bamboo forest area (Li and Lei, 2010), a proportion that is increasing in China and throughout East Asia (Komatsu et al., 2012). Here, we used a rhizome-cut experiment to examine the flow tendency of supplemental water among distinct bamboo culms connected by rhizomes, and to check if the water compensation mechanism among connected culms was a function of night-time water recharge. We also assessed what fraction of night-time water recharge accounted for whole-day water use.

## Study Materials and Methods

### Site Description

This study was conducted in Nankun Mountain Natural Reserve (113°48′–114°51′ E, 23°37′–23°40 N) in Guangdong Province, South China, which supports large areas of *P. pubescens* forests. The mean air temperature in this area is 21.6°C, and the mean annual precipitation is 2,144 mm. The climate of this area is divided into a wet season (April to September) and a dry season (October to March) (Zhang et al., 2017), according to the hydrothermal conditions. Strongly acidic mountainous red soils that are rich in potassium predominate this region (Yang et al., 2013), and, due to the abundant rainfall, the soil water content is relatively high year-round (mean annual value: 33.7 ± 3%) (Zhao et al., 2016). We chose a well-developed stand of *P. pubescens* forest growing on a hill slope, within which we established three experimental plots encompassing a total area of 834 m^2^. The average height of the bamboo in this stand is approximately 15 m, with an average culm diameter at breast height (DBH) of approximately 10.9 ± 1.3 cm, and a standing culm density of 3,600 ha^-1^. No tending or management had been practiced in this bamboo forest in the last 4 years. The characteristics of the bamboo trees in our study site are summarized in **Table 1**.

**Table 1:** Bamboo biometrics.

Age	*A*_s_ (cm^2^)	DBH (cm)	Culm height (m)	*A*_L_ (m^2^)
Juvenile	36.2 ± 3.4	10.2 ± 0.7	13.5 ± 0.6	21.4 ± 6.4
Mature	32.1 ± 1.7	10.4 ± 0.25	13.8 ± 0.2	12 ± 1.9
Senescent	32.8 ± 1.9	10.9 ± 0.29	14.0 ± 0.2	10 ± 1.7

A micrometeorological station was set up in an open site, 50 m away from the experimental plots. The environmental data collected consisted of photosynthetically active radiation (FR3030, Honsea Sunshine Bio Science & Technology Co., Ltd., China), air temperature (T, °C) and relative humidity (Both measured with an HC2-S3, Rotronic, China), and wind speed (FR3120, Honsea Sunshine Bio Science & Technology Co., Ltd., China), all of which were measured automatically every 30 s and recorded at 10-min intervals by a datalogger (DL2e, Delta-T Devices, Ltd., United Kingdom).

### Culm Age Identification

Procedures for identifying the culm ages of *P. pubescens* have been described by Zhao et al. (2016). Culm age is largely determined by the culm color, sheaths and bristles in the sheath ring, and biophilous lichens. The surface of juvenile culms is shiny green, usually covered in a fine white powder, and contains bristles in the sheath ring, with culm sheaths near the ground; only juvenile bamboos have obvious culm sheaths. Mature culms are generally 2–3 years old, yellow (green color beginning to fade), covered in white powder and with fewer bristles, whereas senescent culms often have a mottled whitish appearance, dust on the culm surface and an abundance of attached vegetation, such as lichens. For our study, we chose juvenile bamboos that had reached their maximum height and had photosynthetic leaves.

Data are expressed as the mean ± SD of 15 individual bamboos for each age class (*n* = 15). *A*_s_ is the cross-section area of the bamboo wall, DBH is the diameter at breast height, and *A*_L_ is the whole culm leaf area.

### Sap Flow Measurements

Measurements of sap flow were conducted from December 2012 to December 2013, and then again in July 2014 for a period of 30 days. Three experimental plots were established on the same hillside, and a total of 60 bamboo plants were monitored. In each plot, we selected 15 normal bamboo samples at three age levels and five rhizome-cut samples. Sap flow sensors were installed on 45 normal bamboos and 15 rhizome-cut bamboos.

For each sample culm, one pair of modified, 10-mm long TDP probes was installed at breast height. Two probes were vertically inserted into the culm wall and separated by a node. The heating probe (placed above) was constantly provided with a 120 mA direct current, and the reference probe (placed below) remained unheated. The sap flow density was measured and recorded at 10-min intervals using a Delta-T datalogger. Estimates of sap flow using the standard equation developed by Granier (1987) probably describe the bamboo water use inaccurately because the bamboo hydraulic structure differs significantly from that of the dicot species from which Granier’s original equation was derived; moreover, the parameters used in the original equation were reported to be species-specific (Gao et al., 2016). Thus, we used the calibrated equation described by Zhao et al. (2016), who verified the accuracy of the 10-mm probe technique for measuring sap flow in the *P. pubescens* growing in Nankun Mountain Natural Reserve. This verification was based on an approach involving induced hydraulic pressure and sap-flow changing device, combined with whole-culm pot weighting methods. Finally, the sap flow density (*J*_s_ g H_2_O m^-2^ s^-1^) of *P. pubescens* was calibrated using a modified equation (Zhao et al., 2016):

(1) $$\begin{array}{c} J_{s}=360.44\times {\left(\frac{\Delta Tm-\Delta T}{\Delta T}\right)}^{1.746} \end{array}$$

where ΔTm is the maximum temperature difference obtained under zero sap flow conditions and ΔT is the instantaneous temperature. To account for the possible non-zero sap flow at nights, we applied the ΔTm from the previous as well as the next nights (Litvak et al., 2012) as the baseline. The values 360.44 and 1.746 are the corrected parameters *α* and *β* of the original equation, respectively.

### Rhizome-Cut Experiment

Water and nutrients can potentially be exchanged and allocated among culms via connected rhizomes (Li et al., 2000; Song et al., 2016; Zhao et al., 2016), and these processes would be impeded if the rhizomes were cut. Therefore, a rhizome-cut experiment was conducted in June 2013 to investigate the role of belowground rhizome connections in transpiration and night-time water recharge among culms. We dug narrow trenches around selected culms at a depth of approximately 50 cm to sever the connected rhizomes, and then replaced the soils. Each rhizome-cut culm had a soil surface area of approximately 4 m^2^, and the 15 rhizome-cut bamboos were divided into the three age groups.

### Whole-Plant Hydraulic Conductance

The whole-plant hydraulic conductance (*k*) is an indirect indicator of a tree’s hydraulic structure. Therefore, it can be used to describe the water transport efficiency, which can be expressed as a function of hydraulic conductance in the pathway from soil to leaves (Tyree and Sperry, 1989). According to Schäfer et al. (2000), the whole-plant hydraulic conductance can be expressed as:

(2) $$\begin{array}{c} k=\frac{E_{L}\times A_{L}}{\Psi_{S}-\Psi_{L}-0.001h} \end{array}$$

where *E*_L_ is the sap-flow based canopy transpiration rate (g m^-2^ s^-1^), *k* is the whole-plant hydraulic conductance from the soil to canopy leaves (mmol s^-1^ Mpa^-1^), *Ψ*_s_ is the soil water potential near the roots (MPa), *Ψ*_L_ is the leaf water potential (MPa), *h* is the bamboo height, and 0.01h is the gravity of the water column between the roots and leaves.

We measured the *Ψ*_L_ using a pressure chamber (PMS 1000, Corvallis, OR, United States) on three consecutive fine days, from 05:00 to 17:00 in winter (December 2013) and 05:00 to 19:00 in summer (June 2014), with measurements recorded at 2-h intervals. Nine normal and nine rhizome-cut bamboo culms from each of the three age groups were selected. Three canopy branchlets with 2–3 attached leaves were cut from each sample culm and measured. As the mean annual soil water content in the study site remained high year round (∼33.7 ± 3%), the soil water content was not a limiting factor for daily transpiration in either the wet or the dry season (Zhao et al., 2016). Therefore, as the soil moisture did not vary highly over the course of a day under fine-weather conditions (i.e., in the absence of rainfall), we assumed that the predawn *Ψ*_L_ was equal to *Ψ*_s_ when the sap flow was zero (Ewers et al., 2005). The canopy transpiration rate was calculated as follows:

(3) $$\begin{array}{c} E_{L}=\frac{J_{S\;}\cdot A_{S}}{A_{L}} \end{array}$$

where, *J*_s_ is the mean culm sap flow density (g H_2_O m^-2^ s^-1^) and *A*_L_*/A*_S_ is the ratio of the total leaf to cross-sectional area of the bamboo wall.

Bamboo height and *A*_L_ were determined after the culm harvest, and leaf biomass was converted to leaf area for each sample.

### Night-Time Water Recharge

According to Verbeeck et al. (2007), the night-time water recharge (*W*_n_) is defined as the integrated sum of sap flow density when PAR = 0. The percentage of night-time water recharge to total daily transpiration (*P*, %) was expressed as:

(4) $$\begin{array}{c} P(\%)=\left(\frac{W_{n}}{E}\right)\times 100 \end{array}$$

where, *W*_n_ and *E* are the night-time water recharge and total daily transpiration, respectively. *W*_n_ and *P* were calculated for each age class in both normal and rhizome-cut bamboos.

### Statistical Analyses

December and July represent the dry and wet seasons, respectively, in this study. Oringin Pro 8.5 (OringinLab Corp., United States) was used to simulate the relationship between *W*_n_ and *E* at the different age levels, as well as the relationship between *P* and bamboo morphological features. Statistical analyses of *P* in the wet and dry seasons at the different age levels, and of *P* between bamboo height and age, were performed using a one-way analysis of variance (ANOVA) and covariance analysis, respectively (SPSS 18.0 software, SPSS Inc., Chicago, IL, United States). Correlations between *W*_n_ and *E*, as well as *P* and bamboo morphological features in the wet season were also calculated.

## Results

### Day- and Night-Time Sap Flow in the Wet and Dry Seasons

**FIGURE 1:**
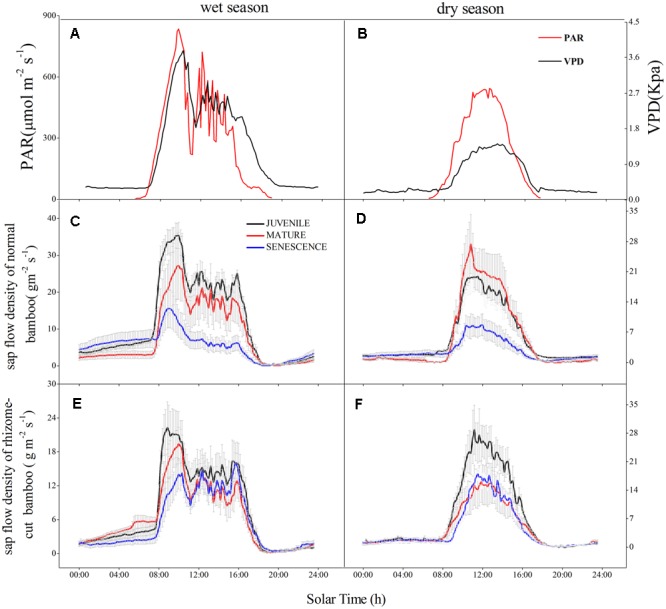
Daily variation of sap flow density (*J*_s_) in three *P. pubescens* individuals in wet (28, 29, 30 July) and dry season (26, 27, 28 December). PAR and VPD in the wet **(A)** and dry **(B)** seasons; normal bamboo in the wet **(C)** and dry **(D)** seasons; rhizome-cut bamboo in the wet **(E)** and dry **(F)** seasons.

**FIGURE 2:**
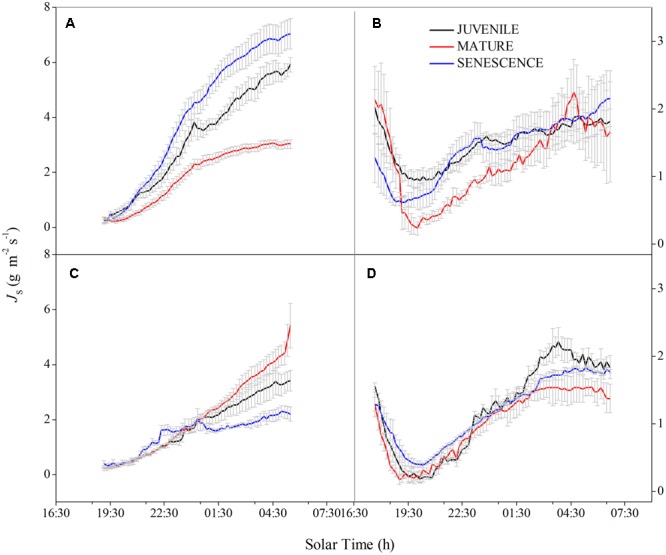
Night-time sap flow density (*J*_s_) pattern. Normal bamboo in wet **(A)** and dry **(B)**. seasons; rhizome-cut bamboo in the wet **(C)** and dry **(D)** seasons. The same day was chosen to describe the pattern as **Figure 1**.

In both wet and dry seasons, the sap flow density (*J*_s_) largely increased after 07:30 due to transpiration and increasing PAR, but decreased to a minimum at 19:00–19:30, then slowly recovered under the action of root pressure (**Figure 1**). The mean daily *J*_s_ of normal and rhizome-cut bamboos in the wet season were 11.55 and 7.31 g m^-2^s^-1^ for juveniles, 8.06 and 6.22 g m^-2^s^-1^ for mature bamboos, and 5.46 and 5.40 g m^-2^s^-1^ for the senescent bamboos, respectively (**Figures 1C,E)**. In the dry season, these values were 5.6 and 7.07 g m^-2^s^-1^ (juvenile), 5.64 and 4.52 g m^-2^s^-1^ (mature), and 2.74 and 4.38 g m^-2^s^-1^ (senescent), respectively (**Figures 1D,F**). Day- and night-time *J*_s_ were higher in the wet season than in the dry season during both day and night (**Figure 2**). In the wet season, the night-time *J*_s_ began at 19:00, and rose over the course of the night to a maximum of 8 g m^-2^ s^-1^ at 06:30, whereas the maximum value was 3 g m^-2^ s^-1^ in the dry season. This part of the integrated sap flow under zero-PAR was considered the effective water recharge in plants, which that compensates for the water deficit induced by day-time transpiration (Zhao, 2010).

### Percentage of Night-Time Water Recharge to Total Daily Transpiration (*P*)

**FIGURE 3:**
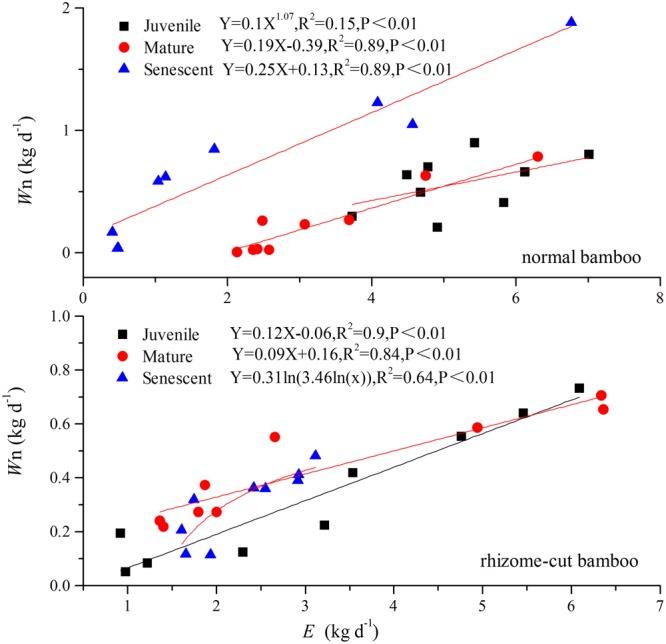
Relationship between night-time water recharge *(Wn)* and total daily transpiration *(E) (n = 9).*

**FIGURE 4:**
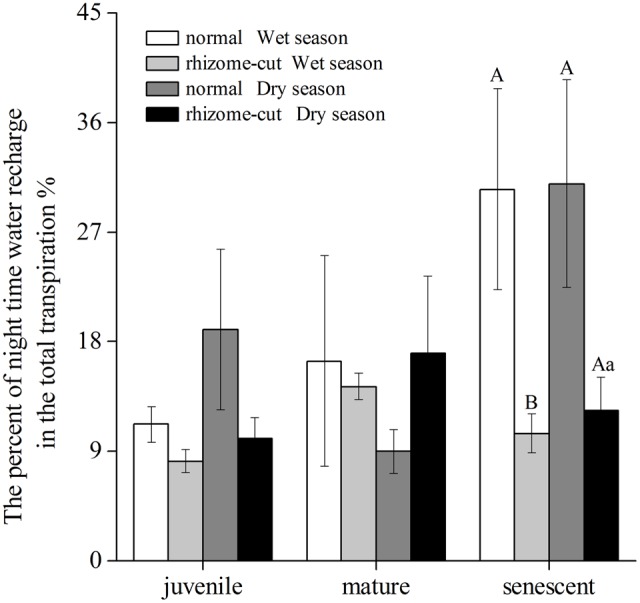
Percentage of night-time water recharge to total daily transpiration *(P)* at different culm ages.

Correlations between *W*_n_ and *E* in the wet season are shown in **Figure 3**. Positive correlations were found between the two factors at all age levels. By comparing the water recharge between normal and rhizome-cut bamboos at different age levels, we found that the fraction of night-time water recharge to total daily transpiration (*P*) was up to 30% during both wet and dry seasons in the normal senescent bamboos, which was higher in comparison with juvenile (11–19%) and mature (9–16%) culms (**Figure 4**). However, *P* in the normal and rhizome-cut senescent bamboos in the wet and dry seasons were 30.83%/10.46% and 30.98%/12.36%, respectively. Values for *P* were significantly lower in the rhizome-cut bamboos than in the normal bamboos because of the significant reduction in night-time sap flow density, as shown in **Figures 2C,D**.

### Whole-Tree Hydraulic Conductance (*k*) and Night-Time Water Recharge

**FIGURE 5:**
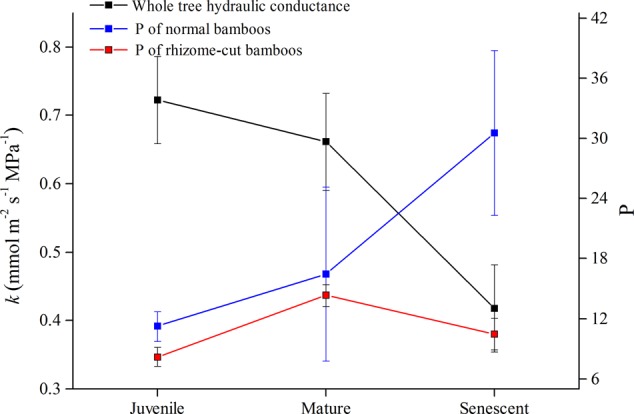
Values for *k* and *P* in normal and rhizome-cut bamboos of different ages.

The whole-tree hydraulic conductance (*k*) significantly decreased with the culm age in normal culms (**Figure 5**), and this was reflected in the lower levels of sap flow density in senescent and rhizome-cut bamboos (**Figure 1**). Interestingly, unlike *k, P* increased with age in normal bamboos (blue line in **Figure 5**). However, *P* in the rhizome-cut bamboos initially trended slightly upward with *k* before decreasing.

### Relationship between *P* and Bamboo Morphological Features

**FIGURE 6:**
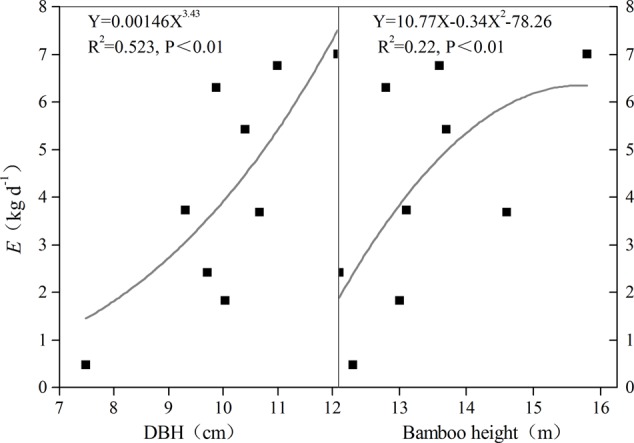
Relationships between mean transpiration *(E)* and DBH and bamboo height (*n = 9*).

**FIGURE 7:**
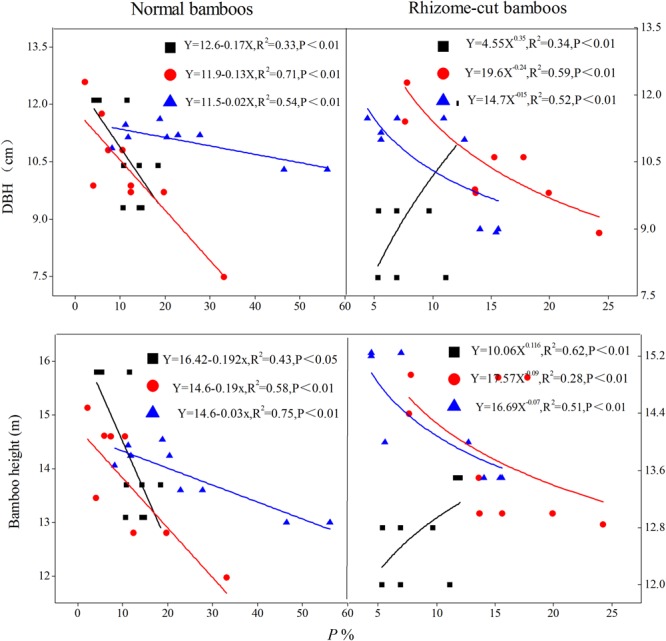
Relationship between *P* and bamboo morphological features. The fitted equations are for juvenile (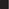), mature (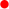), and senescent bamboos (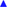) in each pattern, respectively (*n* = 9).

Transpiration (*E*) increased as both culm height and DBH increased; this positive relationship was confirmed through the fitted relationship between *E* and bamboo morphological features (**Figure 6**). However, a negative correlation was observed between *P* and morphological features for night-time water recharge, except in the juveniles of rhizome-cut bamboos (**Figure 7**). Thus, in each age group, taller culms with larger diameters had lower *P* values, indicating that night-time sap flow could not fully replenish the depleted water stores in bigger *P. pubescens* culms.

## Discussion

### Effects of Culm Age and Rhizome on Sap Flow Density

The present work is one of the few studies to quantify the changes in night-time sap flow in bamboos. The sap flow density varied with both culm age and rhizome state. Both day- and night-time *J*_s_ decreased with increasing culm age and after rhizome cutting in the wet season, which showed that the night-time water recharge is affected by culm age and rhizome.

Day-time *J*_s_ and *E* were both lower in older culms (**Figures 1C,E**), whereas night-time *J*_s_ in the wet season did not decline with the bamboo age. Daytime *J*_s_ was inconsistent across the three age levels during the dry season, with mature bamboos having higher *J*_s_ levels than both juvenile and senescent bamboos (**Figure 1D**). This difference was most likely associated with shoot emergence, for which additional water is required. Night-time sap flow fluctuation process coincided with the rhythm of the root pressure activity observed in 59 bamboo species, as reported by Cao et al. (2012). The daytime water absorption was lower in senescent bamboos compared with juvenile and mature bamboos. Senescent bamboos had the weakest water-resource competitiveness and transpiration capacity, owing to reduced hydraulic conductance (**Figure 5)**. Thus, the senescent individuals seemed to rely on night-time *J*_s_ to replenish the water deficit incurred during the day. Moreover, they stored the additional water for transpiration the next day, and even transported it to other individuals in need, indicating that night-time *J*_s_ is more critical to senescent bamboos than the other age groups.

One function of the connected rhizome is to allocate partial water among culms when some of them are experiencing water potential deficits (Zhao et al., 2016). For all bamboo age levels and during both day- and night-time, *J*_s_ was clearly lower in the rhizome-cut bamboos than in the normal bamboos during the wet season, because of the lack of water transference from other culms (Dierick et al., 2010; Zhao et al., 2016). However, *J*_s_ was higher in the rhizome-cut juvenile bamboos than in the normal bamboos (**Figures 1D,F**). Even so, the total transpiration rate of rhizome-cut bamboos was similar to that of normal bamboos (Zhao et al., 2016).

### Night-Time Water Storage in Relation to Morphological Features

*P* was smaller in taller culms with thicker DBH, for three possible reasons. First, water transport paths lengthen and hydraulic resistance increases in taller and older culms (Schulze et al., 1985), leading to less efficient hydraulic conductance (Phillips et al., 2002), stomatal conductance, and photosynthetic capacity (Barnard and Ryan, 2003). Second, taller culms receive more solar radiation than shorter culms, which leads to higher transpiration rates and subsequently greater water uptake by the root system (including self-absorbed and compensation water through connected rhizomes among culms), and therefore, taller culms had a reduced flow of night-time sap in *E*. Finally, positive root pressure was not high enough to drive the night-time sap flow at volumes high enough to satisfy all canopy branches (Cao et al., 2012), and thus, night-time sap flow might only partially recharge the consumed water within leaves, leading to vulnerability segmentation (Zimmermann, 1983). In other words, the plant might elect to protect and hold the water needed by remaining leaves after others wilt or drop (Tyree et al., 1993), which would result in a lower *P* in larger culms.

It is not surprising that positive relationships between morphological features and water use have been observed in many plant species; the larger the tree, the greater is the water consumption (Goldstein et al., 1998; Meinzer et al., 2008). Similar relationships have also been reported for night-time sap flow (Čermák et al., 2004; Takagi, 2013). Here, the covariance analysis revealed that night-time water recharge in *P*. *pubescens* was affected primarily by culm age (*P* = 0.011, α = 0.05), although positive correlations between *E* and morphological features were also observed (**Figure 6**). Zachary (2009) reported that there was no direct correlation between maximum instantaneous sap flow *E* and culm size, but they did detect differences in *E* among various age groups of *Guadua angustifolia*. The positive relationship between *E* and morphological features observed in this study might be due to the little difference in culm height among the *P. pubescens* growing in our study site. In addition, water compensation among culms via connected rhizomes might have weakened the influence of morphological features on night-time water recharge.

### Compensational Adjustment of Hydraulic Conductance in Senescent Culms

In this study, we found that senescent bamboos had distinct water-use characteristics that differed markedly from those of younger culms. Daytime *J*_s_ of senescent bamboos was the lowest among all age groups; however, the night-time *J*_s_ was active in senescent bamboos, and thus, their *P*-value was the highest among all normal culms, which accounted for more than 30% of the total daily transpiration in normal senescent bamboos. *P* was 10.46% and 12.36% in the senescent rhizome-cut bamboos in the wet and dry season, respectively (**Figure 4**), indicating that the soil water content in the study site was not limiting. When the rhizomes were connected, senescent bamboos supplied water to other younger culms (Zhao et al., 2016), but cutting of the rhizomes enabled the senescent culms to reserve more water. Moreover, the night-time water recharge in normal bamboos can largely compensate for the hydraulic limitations resulting from declining whole-tree hydraulic conductance with culm age (Zhao et al., 2016). By comparing the *P* levels of normal and rhizome-cut bamboos, especially in senescent bamboos, we saw the water compensation effect at work in connected culms. As the daytime transpiration increased, the lost water could not be replaced in a timely manner by water from the roots, and therefore, night-time water was needed to replenish the water deficit in culms. The *P* of normal juvenile bamboos accounted for 10% of the total daily water used. Compensation water allocated among culms through connected rhizomes accounts for 20% of the total daily water use in juvenile bamboos (Zhao et al., 2016). Consequently, about 70% of the water consumed during daytime transpiration was acquired via the roots.

Night-time water recharge not only plays an important role in water use and hydraulic regulation, but also indirectly increases the carbon assimilation by relieving the water transport resistance (Gao et al., 2016). To meet the water loss due to excessive leaf transpiration, senescent bamboos could reduce the transpiration by shedding leaves. In the senescent bamboos in our study site, we found that many leaves in lower branches had been dropped, and the total leaf area of senescent bamboos was significantly lower compared with the juvenile culms (*P* = 0.008, α = 0.05). Shedding a few leaves allows the upper canopy of senescent bamboos to achieve higher transpiration and photosynthetic rates in senescent bamboos than in other age group bamboos, as was revealed by the gas-exchange measurements in this bamboo forest (data not shown). This might be a short-term feedback or priming effect to compensate for the leaf shedding (Oren et al., 1999). However, it was only a short-term effect, given that the total daily transpiration of senescent bamboos was still the lowest among all age classes; the total daily transpiration of juvenile, mature, and senescent bamboos was 5.9, 5.6, and 4.4 kg d^-1^, respectively (Zhao et al., 2016). All the above processes were compensatory measures against the reduced whole-tree hydraulic conductance of senescent bamboos. Optimizing the photosynthetic efficiency of the remaining leaves is another means of offsetting the carbon loss due to reductions in leaf area. Thus, night-time water recharge might be a form of insurance to guarantee effective whole-day transpiration. In order to avoid excessive evaporation from the leaves, senescent bamboos can relieve hydraulic limitation through structural and physiological adjustments. Similar to our study, Phillips et al. (2003) noted that water storage significantly affected the water and carbon balance of taller and older trees, and therefore, could partially relieve the hydraulic transport resistance.

### Specific Mechanism of Bamboo Water Transport

Substantial night-time sap flow has been observed in *P. pubescens* individuals. Bamboos are typically shallow-root species, with more than 90% of their root biomass located within the 0–50 cm soil layer (Qiu et al., 1992; Li et al., 1998); such shallow rooting restricts their capacity to tap into the water sources in deeper soil layers. In addition, bamboos tend to have thin stem walls, and thus, only a small area for hydro-active transport, even under good soil moisture conditions. Grass species, including bamboos, therefore, face the problem of frequent cavitation (Holloway-Phillips and Brodribb, 2011). To compensate for these structural failings, vessels in bamboo leaves and the water-conducting conduits in the stem undergo daily cycles of cavitation and water refilling (Yang et al., 2012). Due to the infrequency of stomatal transpiration (stomatal conductance was 8.8 ± 0.05 mmol/m^-2^s^-1^; transpiration rate is 0.06 ± 0.0005 mmol/m^-2^s^-1^) during the night, the presence of active sap flow implied that sap flow was being driven primarily by root pressure as opposed to the capillary pull resulting from transpiration. This stored water can temporarily be used to compensate for the water lost via transpiration during periods of water deficit (Jiang et al., 2004). Flow due to the root pressure can also explain the water recharge and cavitation recovery mechanisms in grasses, although no evidence of cavitation repair through similar mechanisms has been observed in tall conifers, angiosperms (Tyree and Zimmermann, 2002), or other dicots. Studies have also shown that drought-embolized stem recovery in dicots comprises xylem replacement rather than xylem refilling (Brodribb et al., 2010), a repair mechanism that differs considerably from that of bamboo species, which lack the secondary stem growth for water transportation. Considerable parenchyma tissue and lacuna structures within bamboo walls, which act as storage tissues, might represent the evolutionary adaptations for night-time sap flow and water recharge (Liese, 1998; Zachary, 2009); these kinds of structures are useful for the narrow conductive area of culm walls, and can enhance the availability of stored water. Since bamboo species have thin leaves, continuous transpiration would increase the tension within vessels, which could easily generate vessel cavitation in xylem, resulting in xylem hydraulic dysfunction (Cao et al., 2012). Hence, stoma closure and turgor loss in bamboos species are also an adaptive strategy for avoiding water depletion (Yang et al., 2012). We also found that senescent bamboos shed leaves from lower branches, and periodically had both higher transpiration and photosynthetic rates within the leftover leaves, as a positive feedback. The adaptive adjustments and functions controlling water compensation among culms allowed bamboo species to balance water use and carbon accumulation, especially in senescent plants. Efficient night-time water recharge processes not only provide sources of supplementary water to the stores depleted by daily routine plant functioning, but also aid in vessel cavitation repair and the prevention of embolisms (Daley and Phillips, 2006). The lack of xylem tissue regeneration causes the hydraulic conductance and anti-cavitation capabilities to decline with age in the bamboo plants (**Figure 5**). Thus, bamboo species are more dependent on repair mechanisms for the prevention of xylem dysfunction, and consequently, the maintenance of transfusion tissue functioning. The presence of night-time sap flow in bamboos improves water-use efficiency and vessel safety, which are necessary for bamboo vigor, and potentially, survival.

## Conclusion

In this study, we measured sap flow using a self-modified thermal dissipation probe method (TDP, 10 mm rather than the original 20 mm in length) in *P. pubescens* plants of three distinct age groups, in order to calculate the night-time water recharge. Bamboo species have unique night-time water storage requirements because of their physiological and structural characteristics. It was clear that culm age had an effect on the day- and night-time sap flow; moreover, the night-time sap flow in *P. pubescens* was shown to be very active, especially in senescent bamboos. Percentage of the night-time water recharge to total daily transpiration (*P*) was lower in taller culms, suggesting that sap-flow driven by root pressure was limited and could not extend to the highest canopy levels, or to all branches. Belowground, connected rhizomes play a significant role in the water compensation of normal bamboos, and this strongly influenced the night-time water recharge in senescent bamboos; it was required in order to provide sufficient water for whole-day transpiration, because of their weaker whole-tree hydraulic conductance during the daytime. In addition, senescent bamboos dropped leaves and maintained higher rates of transpiration and photosynthesis for short periods of time to avoid hydraulic damage and carbon loss. Water allocation via connected rhizomes and night-time water storage were compensatory mechanisms to offset the hydraulic conductance limitations, and represented important water-resource regulatory strategies for maintaining the normal physiological activity in bamboo.

## Author Contributions

XZ participated in the design of the study, carried out the experiment, collected and analyzed the data, and drafted the manuscript. PZ participated in the design of the study, analysis the data and revision of the manuscript. ZZ, LZ, YH, LO, GN, and QY participated in the design of the study, collection of the data, discussion and interpretation of the results. All authors read and approved the final version of the manuscript.

## Conflict of Interest Statement

The authors declare that the research was conducted in the absence of any commercial or financial relationships that could be construed as a potential conflict of interest.
